# Visualization and Interpretation of Convolutional Neural Network Predictions in Detecting Pneumonia in Pediatric Chest Radiographs

**DOI:** 10.3390/app8101715

**Published:** 2018-09-20

**Authors:** Sivaramakrishnan Rajaraman, Sema Candemir, Incheol Kim, George Thoma, Sameer Antani

**Affiliations:** Lister Hill National Center for Biomedical Communications, National Library of Medicine, Bethesda, MD 20894, USA;

**Keywords:** computer vision, computer-aided diagnosis, convolutional neural networks, pediatric, pneumonia, visualization, explanation, chest X-rays, clinical decision

## Abstract

Pneumonia affects 7% of the global population, resulting in 2 million pediatric deaths every year. Chest X-ray (CXR) analysis is routinely performed to diagnose the disease. Computer-aided diagnostic (CADx) tools aim to supplement decision-making. These tools process the handcrafted and/or convolutional neural network (CNN) extracted image features for visual recognition. However, CNNs are perceived as black boxes since their performance lack explanations. This is a serious bottleneck in applications involving medical screening/diagnosis since poorly interpreted model behavior could adversely affect the clinical decision. In this study, we evaluate, visualize, and explain the performance of customized CNNs to detect pneumonia and further differentiate between bacterial and viral types in pediatric CXRs. We present a novel visualization strategy to localize the region of interest (ROI) that is considered relevant for model predictions across all the inputs that belong to an expected class. We statistically validate the models’ performance toward the underlying tasks. We observe that the customized VGG16 model achieves 96.2% and 93.6% accuracy in detecting the disease and distinguishing between bacterial and viral pneumonia respectively. The model outperforms the state-of-the-art in all performance metrics and demonstrates reduced bias and improved generalization.

## 1. Introduction

**Figure 1.**
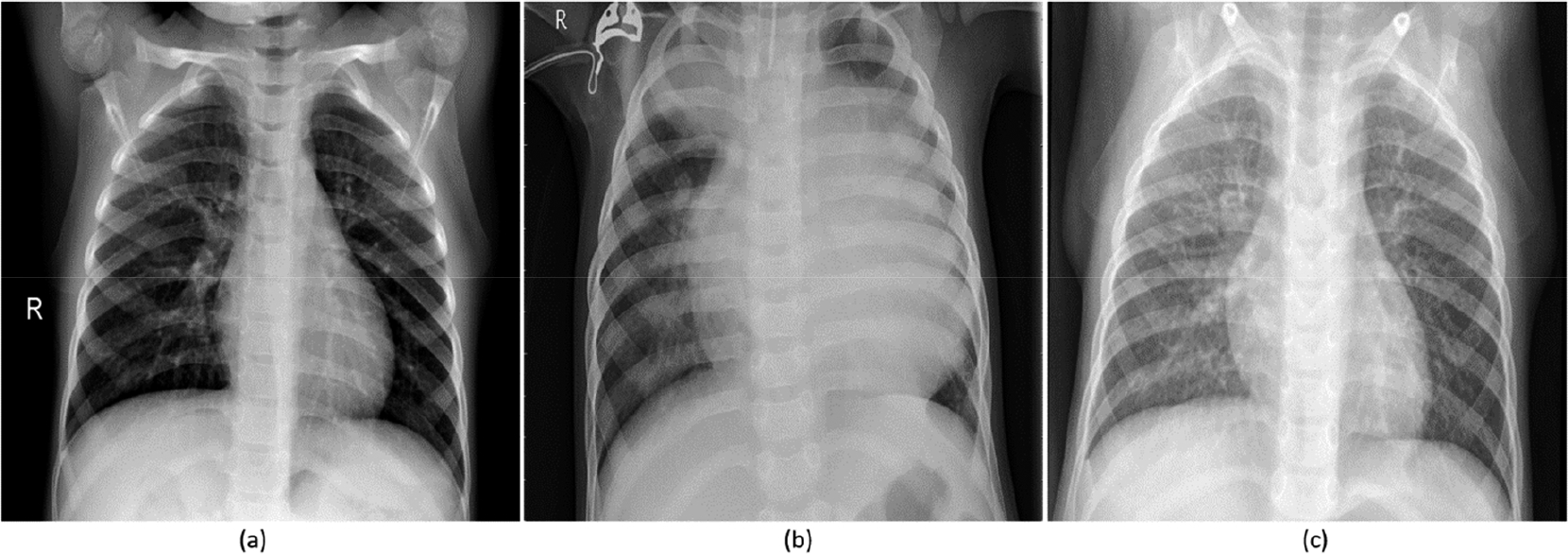
Pediatric CXRs: (**a**) Normal CXR showing clear lungs with no abnormal opacification; (**b**) Bacterial pneumonia exhibiting focal lobar consolidation in the right upper lobe; (**c**) Viral pneumonia manifesting with diffuse interstitial patterns in both lungs.

Pneumonia is a significant cause of mortality in children across the world. According to the World Health Organization (WHO), around 2 million pneumonia-related deaths are reported every year in children under 5 years of age, making it the most significant cause of pediatric death [1]. Pneumonia sourced from bacterial and viral pathogens are the two leading causes and require different forms of management [2]. Bacterial pneumonia is immediately treated with antibiotics while viral pneumonia requires supportive care, making timely and accurate diagnosis important. Chest X-ray (CXR) analysis is the most commonly performed radiographic examination for diagnosing and differentiating the types of pneumonia [3]. However, rapid radiographic diagnoses and treatment are adversely impacted by the lack of expert radiologists in resource-constrained regions where pediatric pneumonia is highly endemic with alarming mortality rates. Figure 1 shows sample instances of normal and infected pediatric CXRs.

Computer-aided diagnostic (CADx) tools aim to supplement clinical decision-making. They combine elements of computer vision and artificial intelligence with radiological image processing for recognizing patterns [4]. Much of the published literature describes machine learning (ML) combine elements of computer vision and artificial intelligence with radiological image processing for recognizing patterns [4]. Much of the published literature describes machine learning (ML) algorithms that use handcrafted feature descriptors [5] that are optimized for individual datasets and trained for specific variability in size, orientation, and position of the region of interest (ROI) [6]. In recent years, data-driven deep learning (DL) methods are shown to avoid the issues with handcrafted features through end-to-end feature extraction and classification.

Convolutional neural networks (CNNs) belong to a class of DL models that are prominently used in computer vision [7]. These models have multiple processing layers to learn hierarchical feature representations from the input pixel data. The features in the early network layers are abstracted through the mechanisms of local receptive fields, weight sharing, and pooling to form rich feature representations toward learning and classifying the inputs to their respective classes. Due to lack of sufficiently extensive medical image data, CNNs trained on large-scale data collections such as ImageNet [8] are used to transfer the knowledge of learned representations in the form of generic image features to the current task. CNNs are also shown to deliver promising results in object detection and localization tasks [9].

The astounding success of deep CNNs coupled with lack of explainable decision-making has resulted in a perception of doubt. This poorly understood model behavior has limited their use in routine clinical practice [10]. There aren’t enough studies pertaining to the visualization and interpretation of CNNs in medical image analysis/understanding applications. In this article, we (i) detect and distinguish pneumonia ty pes in pediatric CXRs, and (ii) explain the internal operations and predictions of CNNs applied to this challenge.

In this study, we evaluate, visualize, and explain the predictions of CNN models in classifying pediatric CXRs to detect pneumonia and furthermor e to differentiate between bacterial and viral pneumonia to facilitate swift referrals that require urgent medical intervention. We propose a novel method to visualize the class-specific ROI that is considered significant for correct predictions across all the inputs that belong to an expected class. We evaluate and statistically validate the performance of different customized CNNs that is trained end to-end on the dataset under study to provide an accurate and timely diagnosis of the pathology. The work is organized as follows: Section 2 discusses the related work, Section 3 elaborates on the materi als and methods, Section 4 discusses the results, and Section 5 concludes the study.

## 2. Related Work

A study of the literature reveals several works pertaining to the use of handcrafted features for detecting pneumonia in chest radiographs [11–14]. However, few studies reported the performance of DL methods applied to pneumonia detection in pediatric CXRs. Relatively few researchers attempted to offer a qualitative explanation of their model’s learned behavior, internal computations, and predictions. The authors of [15] used a pretrained InceptionV3 model as a fixed feature extractor to classify normal and pneumonia-infected pediatric CXRs and further distinguish between bacterial and viral pneumonia with an area under the curve (AUC) of 0.968 and 0.940 respectively. In another study [4], the authors used a gradient-based ROI localization algorithm to detect and spatially locate pneumonia in CXRs. They released the largest collection of the National Institutes of Health (NIH) CXR dataset that contains 112,120 frontal CXRs, the associated labels are text-mined from radiological reports using natural language processing tools. The authors reported an AUC of 0.633 toward detecting the disease. The authors of [16] used a gradient-based visualization method to localize the ROI with heat maps toward pneumonia detection. They used a 121-layer densely connected neural network toward estimating the disease probability and obtained an AUC of 0.768 toward detecting pneumonia. The authors of [17] used an attention-guided mask inference algorithm to locate salient image regions that stand indicative of pneumonia. The features of local and global network branches in the proposed model are concatenated to estimate the probability of the disease. An AUC of 0.776 is reported for pneumonia detection.

## 3. Materials and Methods

### 3.1. Data Collection and Preprocessing

We used a set of pediatric CXRs that have been made publicly available by the authors of [15]. The authors have obtained approvals from the Institutional Review Board (IRB) and Ethics Committee toward data collection and experimentation. The dataset includes anteroposterior CXRs of children from 1 to 5 years of age collected from Guangzhou Women and Children’s Medical Center in Guangzhou, China. The characteristics of the data and its distribution are shown in Table 1. The dataset is screened for quality control to remove unreadable and low-quality radiographs and curated by experts to avoid grading errors.

**Figure 2.**
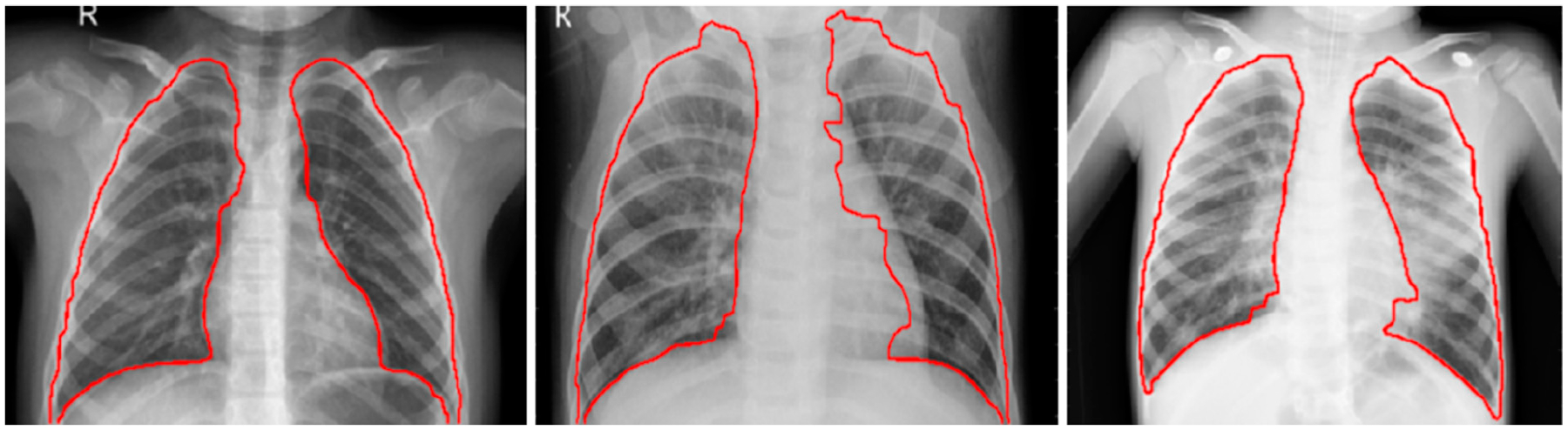
Detected boundaries in sample pediatric CXRs.

The CXRs contain regions other than the lungs that do not contribute to diagnosing pneumonia. Under these circumstances, the model may learn irrelevant feature representations from the underlying data. Using an algorithm based on anatomical atlases [18] to automatically detect the lung ROI can avoid this. A reference set of patient CXRs with expert-delineated lung masks are used as models [19] to register with the objective pediatric CXR. When presented with an objective chest radiograph, the algorithm uses the Bhattacharyya distance measure to select the most similar model CXRs. The correspondence between the model CXRs and objective CXR is computed by modeling the objective CXR with local image feature representations and identifying similar locations by applying SIFT-flow algorithm [20]. This map is the transformation applied to the model lung masks to transform them into the approximate lung model for the objective chest radiograph. The lung boundaries are cropped to the size of a bounding box to include all the lung pixels that constitute the ROI for the current task. The baseline data (whole CXRs) and the cropped bounding box are resampled to 1024 × 1024 pixel dimensions and mean normalized to assist the models in faster convergence. The detected lung boundaries for the sample pediatric CXRs are shown in Figure 2.

### 3.2. Configuring CNNs for Pneumonia Detection

We evaluated the performance of different customized CNNs and a VGG16 model in detecting pneumonia and furthermore distinguishing between bacterial and viral types to facilitate timely and accurate disease diagnosis. We evaluated the performance of three different customized CNN architectures: (i) Sequential CNN; (ii) CNN with residual connections (Residual CNN); and, (iii) CNN with Inception modules (Inception CNN).

#### 3.2.1. Sequential CNN

**Figure 3.**
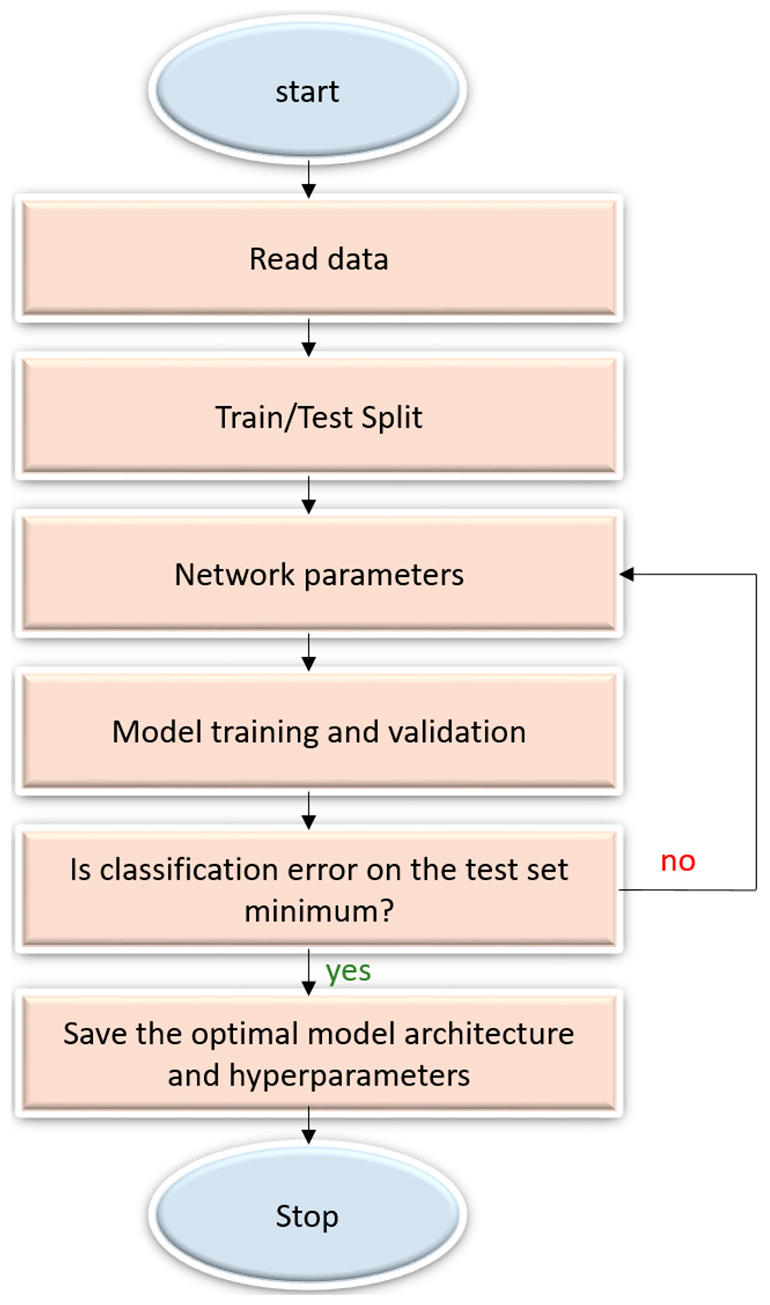
Flowchart describing the optimization procedure.

A sequential CNN model belongs to the class of deep, feed-forward artificial neural networks that are commonly applied to visual recognition [7]. It is a linear stack of convolutional, nonlinear, pooling, and dense layers. We optimized the sequential CNN architecture and its hyperparameters for the datasets under study through Bayesian learning [21,22]. The procedure uses a Gaussian process model of an objective function and its evaluation to optimize the network depth, learning rate, momentum, and L2-regularization. These parameters are passed as arguments in the form of optimization variables to evaluate the objective function. We initialized the search ranges to [110], [1 × 10^−7^ 1 × 10^−1^], [0.7 0.99], and [1 × 10^−10^ 1 × 10^−2^] for the network depth, learning rate, momentum, and L2-regularization respectively. The objective function takes these variables as input, trains, validates and saves the optimal network that gives the minimum classification error on the test data. Figure 3 illustrates the steps involved in optimization.

#### 3.2.2. Residual CNN

**Figure 4.**
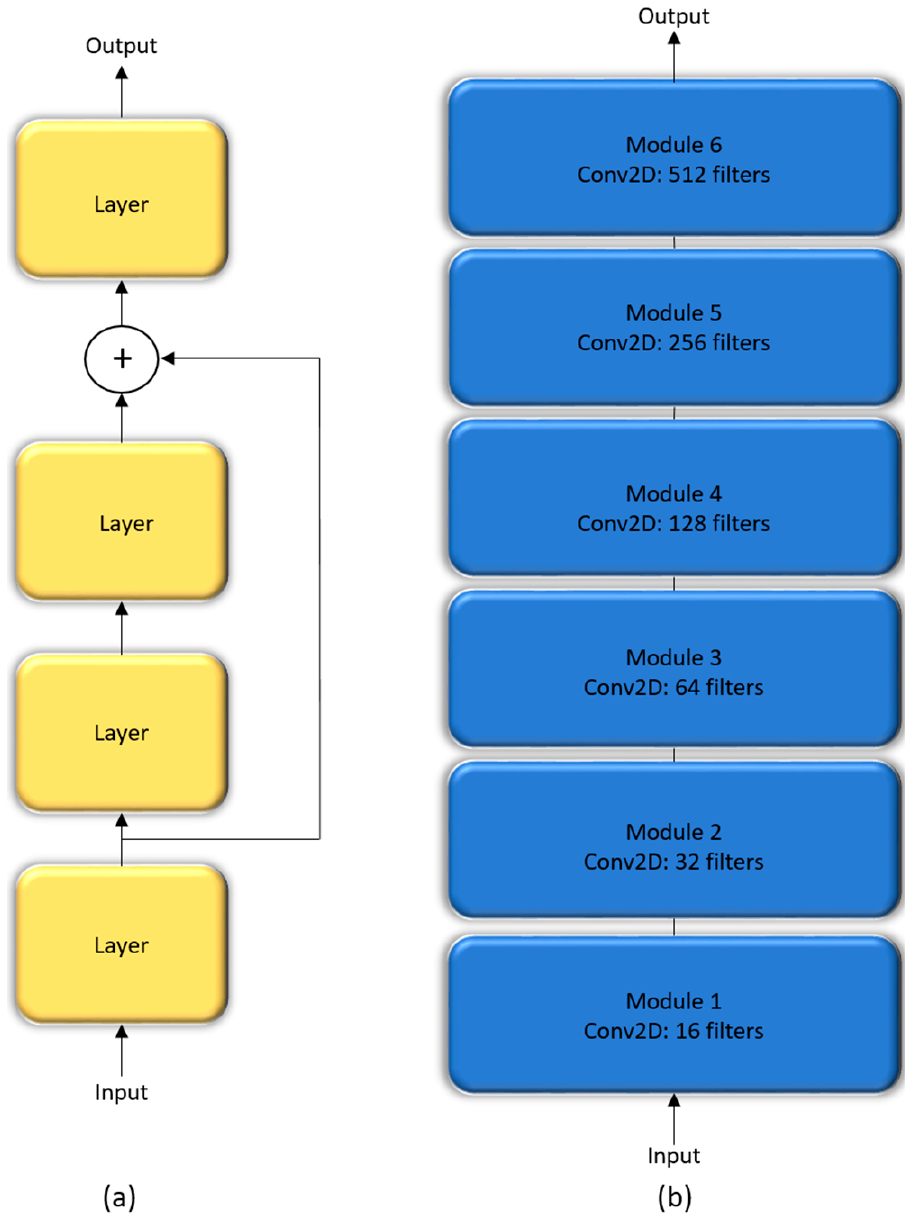
The architecture of customized residual CNN: (**a**) Residual block; (**b**) Customized residual CNN stacked with six residual blocks.

In a sequential CNN, the succeeding network layer learns the feature representations from only the preceding layer. These networks are constraine d by the level of information they can process. Residual networks are proposed by [23] that wo n the ImageNet Large Sc ale Visual Recognition (ILSVRC) Challenge in 2015. These networks tackle the issue of representational bottlenecks by injecting the information from the earlier network layers downstream to prevent loss of information. They also prevent the gradients from vanishing by introducing a linear information carry track to propagate gradients through deep network layers. In this study, we propose a customized CNN that is made up of six residual blocks, as shown in Figure 4.

#### 3.2.3. Inception CNN

**Figure 5.**
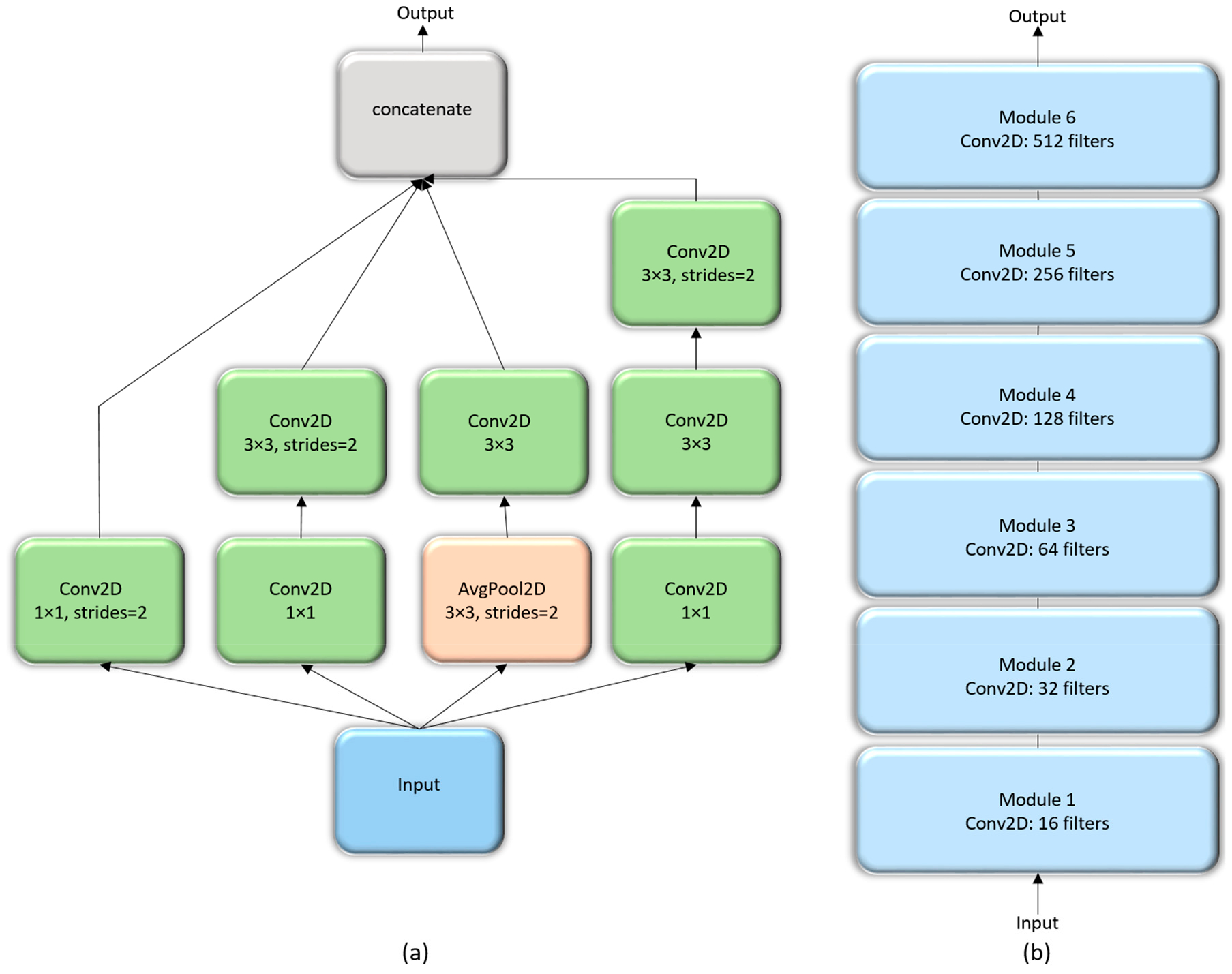
The architecture of customized InceptionV3 CNN: (**a**) InceptionV3 module; (**b**) Customized Inception CNN stacked with six InceptionV3 modules.

The Inception architecture, proposed by [24] consists of independent modules having parallel branches that are concatenated to form the resultant feature map that is fed into the succeeding modules. Unlike sequential CNN, this method of stacking modules help in separately learning the spatial and channel-wise feature representations. The 1 × 1 convolution filters used in these modules factor out the channel and spatial feature learning by computing features from the channels without mixing spatial information by looking at one input tile at a given point in time. We construct a customized Inception CNN by stacking six InceptionV3 modules [23], as shown in Figure 5.

#### 3.2.4. Customized VGG16

**Figure 6.**
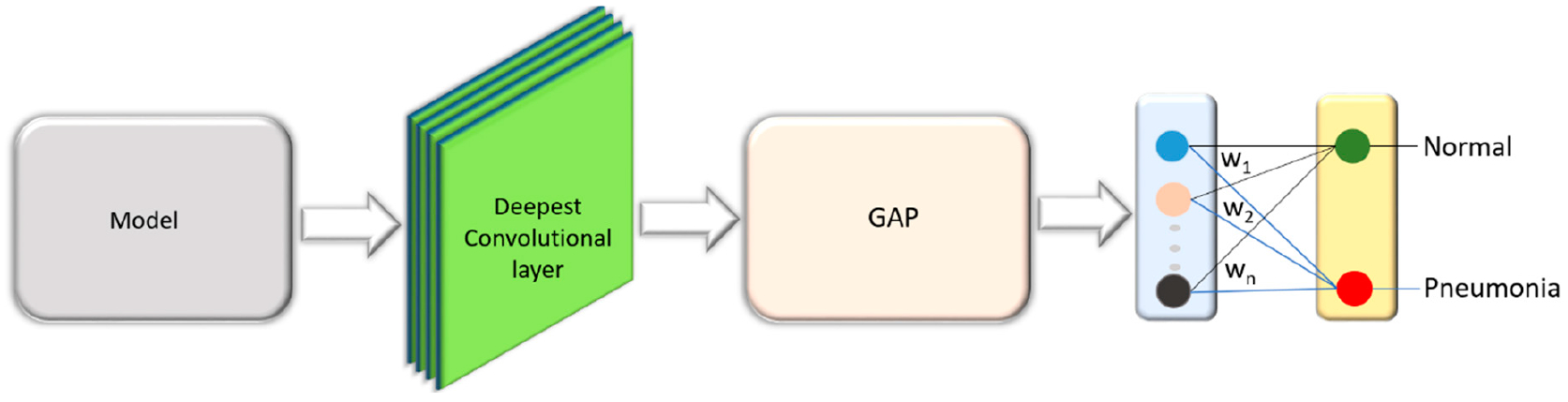
VGG16 model truncated at the deepest convolutional layer and added with a GAP and dense layer.

VGG16 is proposed and trained by the Oxford’s Visual Geometry Group (VGG) [25] for object recognition. The model scored first in ILSVRC image localization and second in image classification tasks. We customized the architecture of VGG16 model and evaluated its performance toward the tasks of interest. The model is truncated at the deepest convolutional layer and added with a global average pooling (GAP) and dense layer as shown in Figure 6. We refer to this model as customized VGG16 in this study.

The hyperparameters of the customized residual, Inception and VGG16 models are optimized through a randomized grid search [26] that searches and optimizes the value of hyperparameters including learning rate, momentum, and L2-regularization. The search ranges are initialized to [1 × 10^−6^ 1 × 10^−1^], [0.7 0.99], and [1 × 10^−10^ 1 × 10^−1^] for the learning rate, momentum, and L2-regularization respectively. Callbacks are used to view the internal states during training and retain the best performing model for analysis. We performed hold-out testing with the test data after every step. The performance of customized CNNs are evaluated in terms of the following performance metrics: (i) accuracy; (ii) AUC; (iii) precision; (iv) recall; (v) specificity; (vi) F-Score; and, (vii) Matthews Correlation Coefficient (MCC).We used the NIH Biowulf Linux cluster (https://hpc.nih.gov/) and the high performance computing facility at the National Library of Medicine (NLM) for computational analyses. Software frameworks included with Matlab R2017b are used to configure and evaluate the sequential CNN along with Keras and Tensorflow backend for other customized models used in this study.

### 3.3. Visualization Studies

The interpretation and understanding of CNNs is a hotly debated topic in ML, particularly in the context of clinical decision-making [4]. CNNs are perceived as black boxes and it is imperative to explain their working to build trust in their predictions [9]. This helps to understand their working principles, assist in hyperparameter tuning and optimization, identify and get an intuition of the reason behind the model failures, and explain the predictions to the end-user prior to possible deployment. The methods of visualizing CNNs are broadly categorized into (i) preliminary methods that help to visualize the overall structure of the model; and, (ii) gradient-based methods that manipulate the gradients from the forward and backward pass during training [27]. We demonstrated the overall structure of the CNNs, as shown in Figures 4–6.

#### 3.3.1. Visual Explanation through Discriminative Localization

The trained model focusses on discriminative parts of the image to arrive at the predictions. Class Activation Maps (CAM) help in visualizing and debugging model predictions, particularly in case of a prediction error when the model predicts based on the surrounding context [27]. The output of the GAP layer is fed to the dense layer to identify the discriminative ROI localized to classify the inputs to their respective classes. Let *G*^*m*^ denote the GAP that spatially averages the *m*-th feature map from the deepest convolutional layer, and $w_{m}^{p}$ denote the weights connecting the *m*-th feature map to the output neuron corresponding to the expected class p. A prediction score *S*_*p*_ at the output neuron is expressed as a weighted sum of GAP as shown in Equation (1).

(1) $$S_{p}=\sum_{m}w_{m}^{p}\sum_{x,y}g_{m}\left(x,y\right)=\sum_{x,y}\sum_{m}w_{m}^{p}g_{m}\left(x,y\right)$$

The value *g*_*m*_ (*x*, *y*) denotes the *m*-th feature map activation in the spatial location (*x*, *y*). The CAM for the class *p* denoted by *CAM*_*p*_ is expressed as the weighted sum of the activations from all the feature maps with respect to the expected class *p* at spatial location (*x*, *y*) as shown in Equation (2).

(2) $$CAM_{p}\left(x,y\right)=\sum_{m}w_{m}^{p}g_{m}\left(x,y\right)$$

CAM gives information pertaining to the importance of the activations at each spatial grid (*x*, *y*) to It is rescaled to the size of the input image to classify an input image to its expected class *p*. It is rescaled to the size of the input image to locate the discriminative ROI used to classify the image to its expected class. This helps to answer queries pertaining to the ability of the model in predicting and localizing the ROI specific to its category. We propose a novel visualization method called average-CAM to represent the class-level ROI that is most commonly considered significant across for correct all class. prediction the inputs that belong to a given class. The average-CAM for the class *p* is computed by averaging the CAM outputs as shown in Equation (3).

(3) $$\text{average}-CAM_{p}\left(x,y\right)=\sum_{a}CAM_{p}^{a}\left(x,y\right)$$

$CAM_{p}^{a}\left(x,y\right)$ denotes the CAM for the *a*-th image in the expected class *p*. This helps to identify the ROI specific to the expected class, improve the interpretability of the internal representations, and explainability of the model predictions.

CAM visualization can only be applied to networks with a GAP layer. Gradient-weighted CAM (grad-CAM) is a strict generalization of CAM that can be applied to all existing CNNs [28]. It uses the gradient information of the expected class, flowing back into the deepest convolutional layer to generate explanations. Grad-CAM produces the weighted sum of all the feature maps in the deepest convolutional layer for the expected class *p* as shown in Equation (4). A ReLU nonlinearity is applied to avoid the negative weights from influencing the class *p*. This is based on the consideration that the pixels with negative weights are likely to belong to other classes.

(4) $$\text{grad}-CAM_{p}\left(x,y\right)=\text{ReLU}\left(\sum_{m}\beta_{m}^{p}g_{m}\left(x,y\right)\right)$$

The value $\beta_{m}^{p}$ is obtained by computing the gradient of the prediction score *S*_*p*_ with respect to the *m*-th feature map as shown in Equation (5).

(5) $$\beta_{m}^{p}=\sum_{x,y}\frac{\partial S_{p}}{\partial g_{m}\left(x,y\right)}$$

According to Equations (1) and (4), $\beta_{m}^{p}$ is precisely the same as $w_{m}^{p}$ for networks with a CAM-compatible architecture. The difference lies in applying the ReLU non-linearity to exclude the influence of negative weights that are likely to belong to other classes. The average-grad-CAM for the class *p* is computed by averaging the grad-CAM outputs as shown in Equation (6). The value $\text{grad-}CAM_{p}^{a}\left(x,y\right)$ denotes the grad-CAM for the *a-*th image in the expected class *p*.

(6) $$\text{average}-\text{grad}-CAM_{p}\left(x,y\right)=\underset{a}{\sum }\text{grad}-CAM_{p}^{a}\left(x,y\right)$$

#### 3.3.2. Model-Agnostic Visual Explanations

Local interpretable model-agnostic explanations (LIME) is a visualization tool proposed by [29]. It helps to provide a qualitative interpretation of the relationship between perturbed input instances and the model predictions. The input image is divided into contiguous superpixels and a dataset of perturbed input instances is constructed by turning on/off these interpretable components. The perturbed instances are weighted by their similarity to the explained instance. The algorithm approximates the CNN by a sparse, linear model that is weighted only in the neighborhood of the explained predictions. An explanation is generated in the form of superpixels with the highest positive weights that demonstrate the discriminative ROI localized by the model to classify the image to its expected class. Let $k\in {\mathbb{R}}^{d}$ be the explained instance, and *k’* ∈ {0, 1}^*d*^, the binary vector that denotes the presence/absence of a superpixel. Let *g* ∈ *G* denote the explanation where *G* is a class of interpretable linear models. Let γ(*g*) denote the complexity measure associated with the explanation *g* ∈ *G*. The value γ(*g*) denotes the number of non-zero coefficients for the linear model. Let $m:{\mathbb{R}}^{d}\to \mathbb{R}$ denote the explained model and *m*(*k*), the probability that *k* belongs to a given class. Let Π_*k*_(*x*) denote the measure of proximity between the instance *x* to *k* and *P*(*m*, *g*, Π_*k*_) denote the loss of *g* toward approximating *m* in the neighborhood defined by Π_*k*_. The value *P*(*m*, *g*, Π_*k*_) is minimized and the value of γ(*g*) remains low enough for interpretability. Equation (7) gives the explanations produced by LIME.

(7) $$\beta (k)=\underset{g\in G}{\text{argmin}}P\left(m,g,\prod_{k}\right)+\gamma (g)$$

The value *P* (*m*, *g*, Π_*k*_) is approximated by drawing samples weighted by Π_*k*_. Equation (8) shows an exponential kernel defined on the L2-distance function (J) with width €. For a given input perturbed sample *b’* ∈ {0, 1}^*d’*^ containing a fraction of non-zero elements, the label for the explanation model *m*(*b*) is obtained by recovering the sample in the original representation $b\in {\mathbb{R}}^{d}$ as shown in Equation (9).

(8) $$\prod_{k}^{b}=\text{exp}\left(-J\frac{{\left(y,b\right)}^{2}}{\epsilon^{2}}\right)$$

(9) $$P\left(m,g,\prod_{k}\right)=\sum_{b,b\in B}\prod_{k}^{b}{\left(m\left(b\right)-g\left(b^{\prime }\right)\right)}^{2}$$

LIME provides explanations that help to make an informed decision about the trustworthiness of the predictions and gain crucial insights into the model behavior.

## 4. Results and Discussion

### 4.1. Performance Evaluation of Customized CNNs

**Figure 7.**
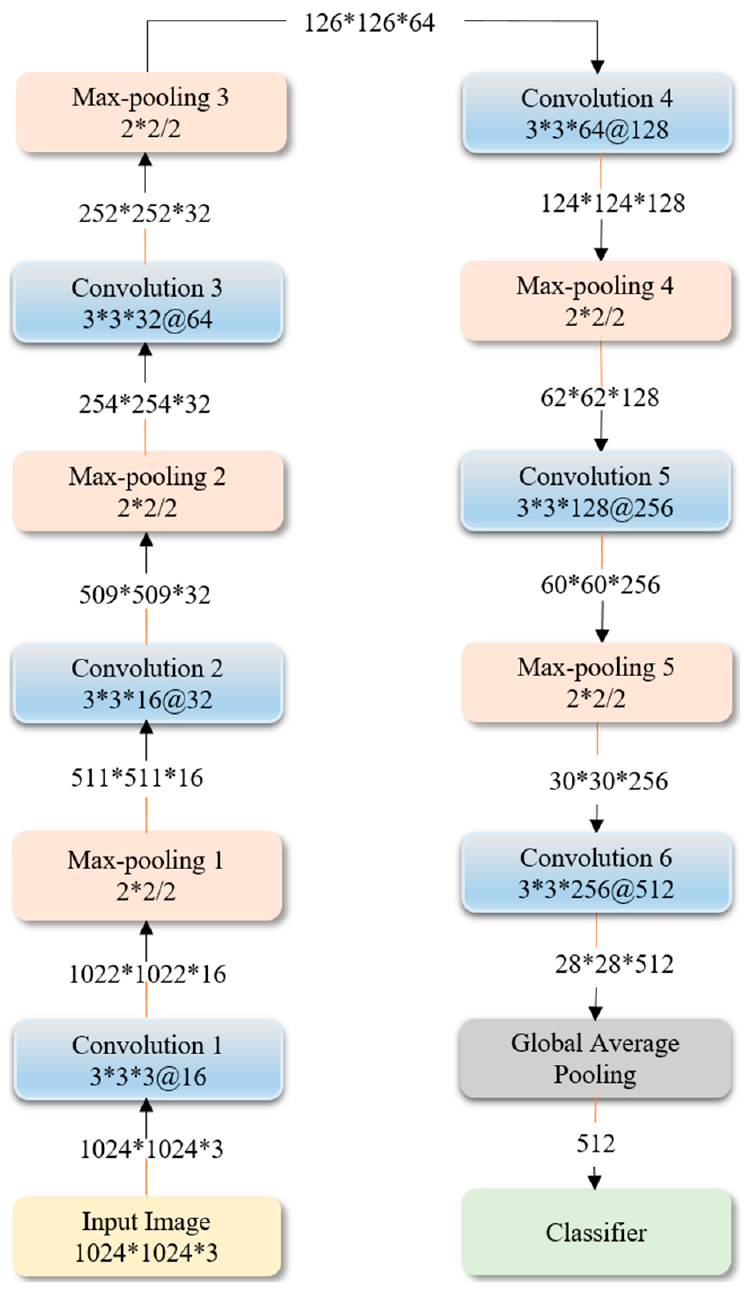
The optimized architecture of customized sequential CNN.

Figure 7 shows the optimized architecture and parameters of the sequential CNN, obtained through Bayesian learning. We performed 100 objective function evaluations toward optimizing the model parameters. The optimized values are found to be 6, 1 × 10^−3^, 0.9, and 1 × 10^−6^ for the network depth, learning rate, momentum, and L2-regularization parameters respectively. The number of convolutional layer filters is increased by a factor of 2 each time amax-pooling layer is used, in order to ensure roughly the same number of computations in the network layers. Rectified Linear Unit (ReLU) layers are added to introduce non-linearity and prevent vanishing gradients during backpropagation [7].

Our analysis shows an increase in the performance of the residual and inception CNNs when the number of filters in the convolutional layers of the succeeding blocks are increased by a factor of 2. We found the optimal hyperparameter values for the residual, inception, and VGG16 models through a randomized grid search. The values are tabulated in Table 2.

The customized CNNs are evaluated with the baseline and cropped ROI data. The results are tabulated in Table 3. We observed that the performance of the models with the cropped ROI is relatively promising in comparison to the baseline in classifying normal and pneumonia infected CXRs. This is obvious because the models trained with the cropped ROI learn relevant feature representations toward classifying the task of interest.

**Figure 8.**
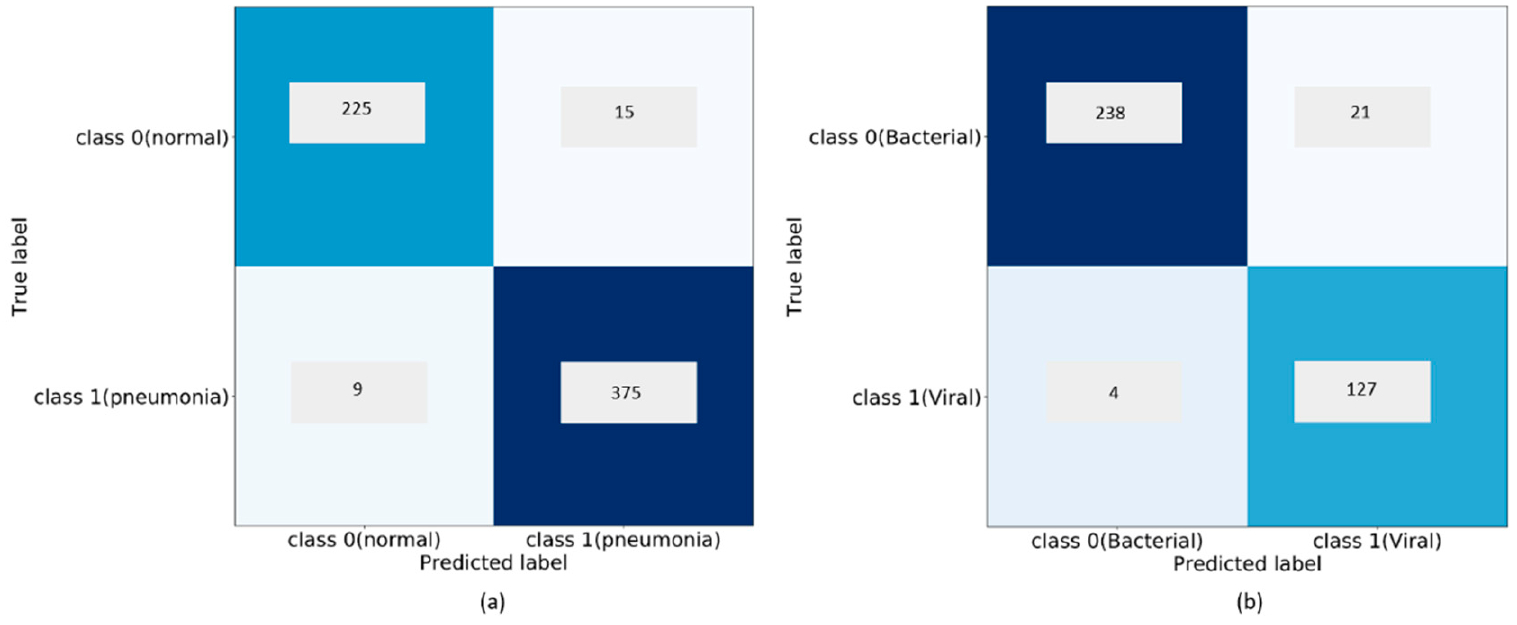
Confusion matrices for the performance of the customized VGG16 model: (**a**) Normal v. Pneumonia; (**b**) Bacterial v. Viral Pneumonia.

**Figure 9.**
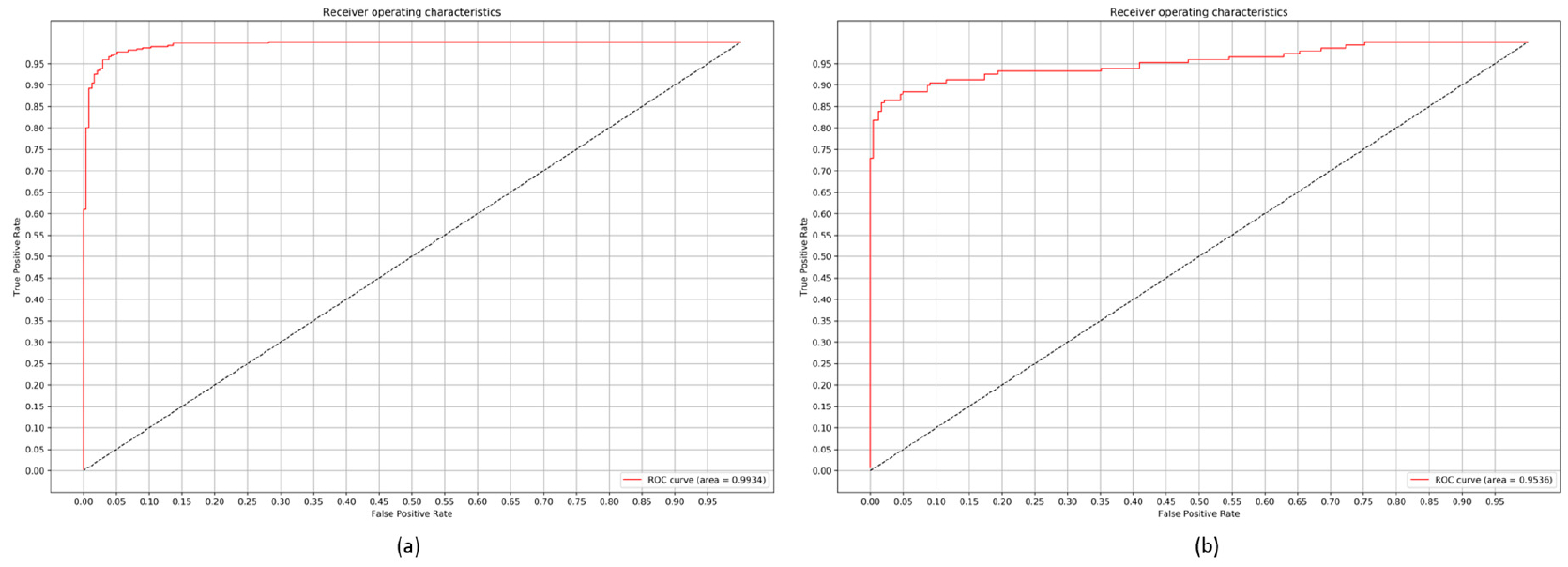
ROC curves demonstrating the performance of the customized VGG16 model: (**a**) Normal v. Pneumonia; (**b**) Bacterial v. Viral Pneumonia.

**Figure 10.**
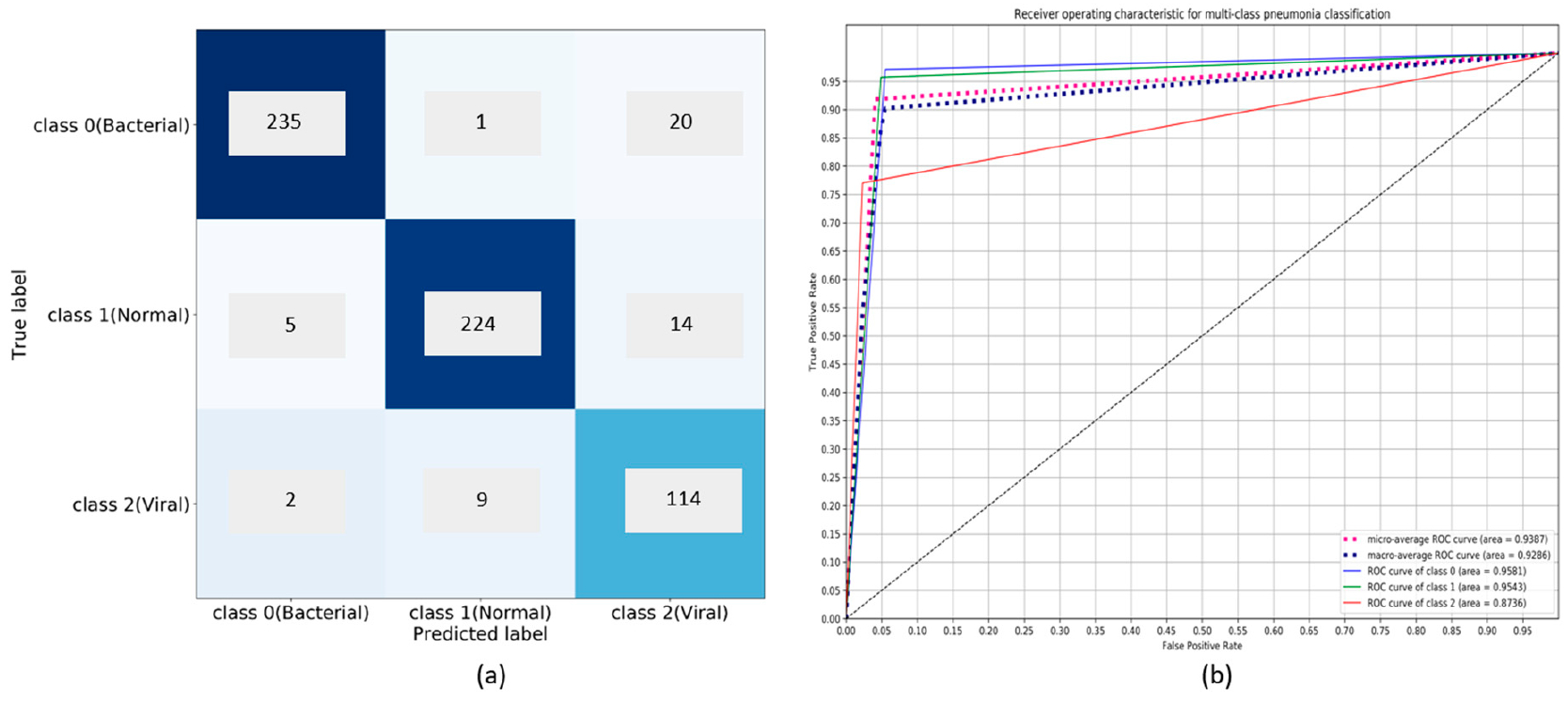
Performance of customized VGG16 model in multiclass classification: (**a**) Confusion matrix; (**b**) ROC curves.

The customized VGG16 model demonstrates promising performance than the other CNNs under study. The model learned generic image features from ImageNet that served as a good initialization compared to random weights and trained end-to-end on the current tasks to learn task-specific features. This results in faster convergence with reduced bias, overfitting, and improved generalization. In classifying bacterial and viral pneumonia, no significant difference in performance is observed for the customized VGG16 model with the baseline and cropped ROI. In the multi-class classification task, the cropped ROI gave better results than the baseline data. However, we observed that the differences in performance are not significant. This may be due to the reason that the dataset under study already appeared as cropped, and the boundary detection algorithm resulted in a few under-segmented regions near the costophrenic angle. The customized sequential, residual, and inception CNNs with random weight initializations didn’t have the opportunity to learn discriminative features, owing to the sparse availability and imbalanced distribution of training data across the expected classes. We observed that the sequential CNN outperformed the residual and inception counterparts across the classification tasks. The usage of residual connections is beneficial in resolving the issue of representational bottlenecks and vanishing gradients in deep models. The CNNs used in this study have a shallow architecture. The residual connections did not introduce significant gains into the performance for the tasks of interest. Unlike ImageNet, the variability in the pediatric CXR data is several orders of magnitude smaller. The architecture of residual and inception CNNs are progressively more complex and did not seem to be a fitting tool to use for the tasks of interest. The confusion matrices and AUC achieved with the customized VGG16 model are shown in Figures 8–10. We observed that the training metrics are poor compared to test accuracy. This is due to the fact that noisy images are included in the training data to reduce bias, overfitting, and improve model generalization.

We compared the performance of the customized VGG16 model trained with the cropped ROI, to the state-of-the-art. The results are tabulated in Table 4. We observed that our model outperforms the current literature in all performance metrics across the classification tasks. The customized sequential CNN demonstrates higher values for recall in: (i) classifying normal and pneumonia; and, (ii) identical recall measures to the customized VGG16 model in classifying bacterial and viral pneumonia. However, considering the balance between precision and recall as demonstrated by the F-Score and MCC, the customized VGG16 model outperforms the other CNNs and the state-of-the-art across the classification tasks.

### 4.2. Visualization Discriminative Localization through

**Figure 11.**
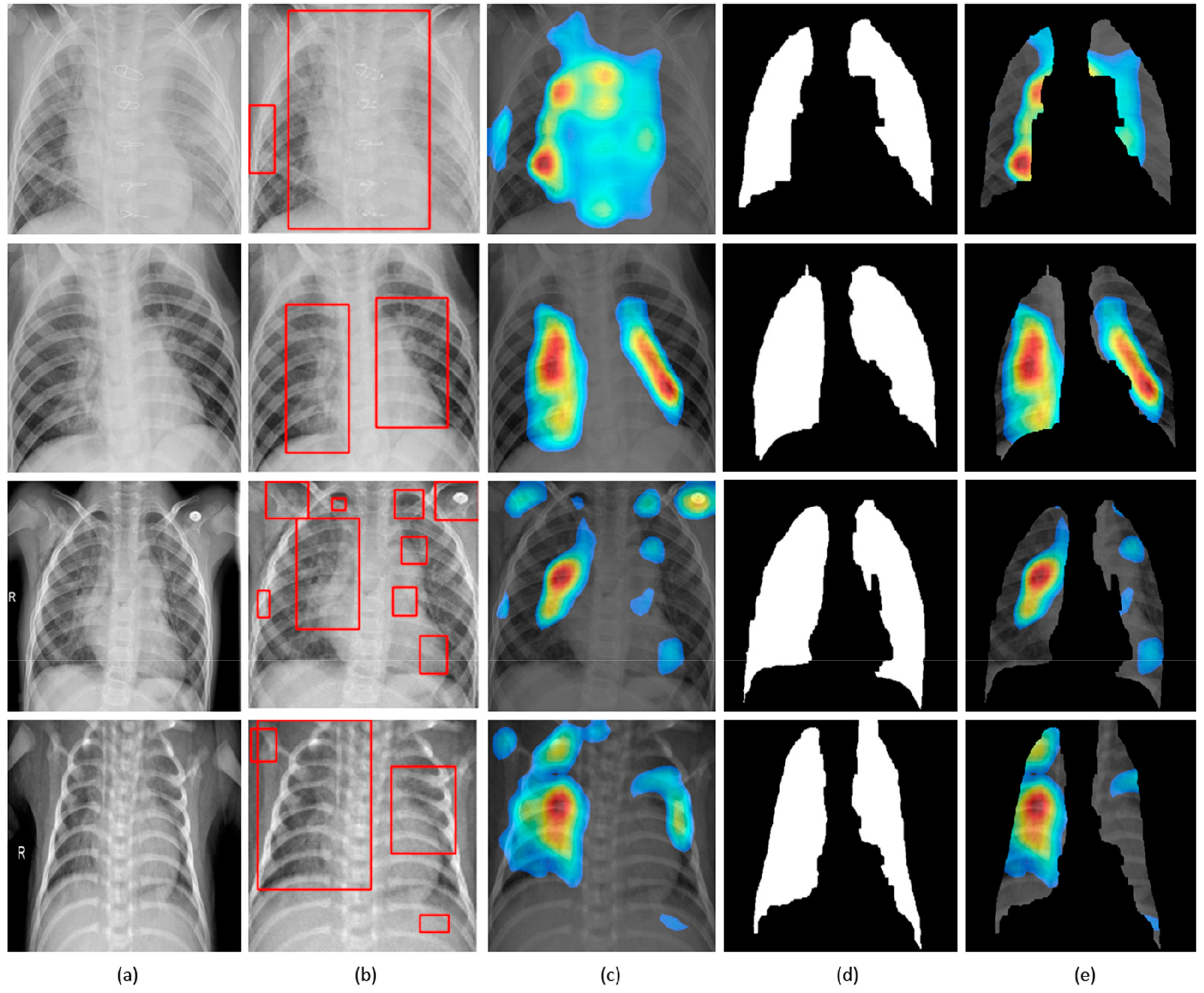
Visual explanations through gradient-based localization using CAM: (**a**) Input CXRs; (**b**) Bounding boxes localizing regions of activations; (**c**) CAM showing heat maps superimposed on the original CXRs; (**d**) Automatically segmented lung masks; (**e**) CAM showing heat maps superimposed on the cropped lungs.

**Figure 12.**
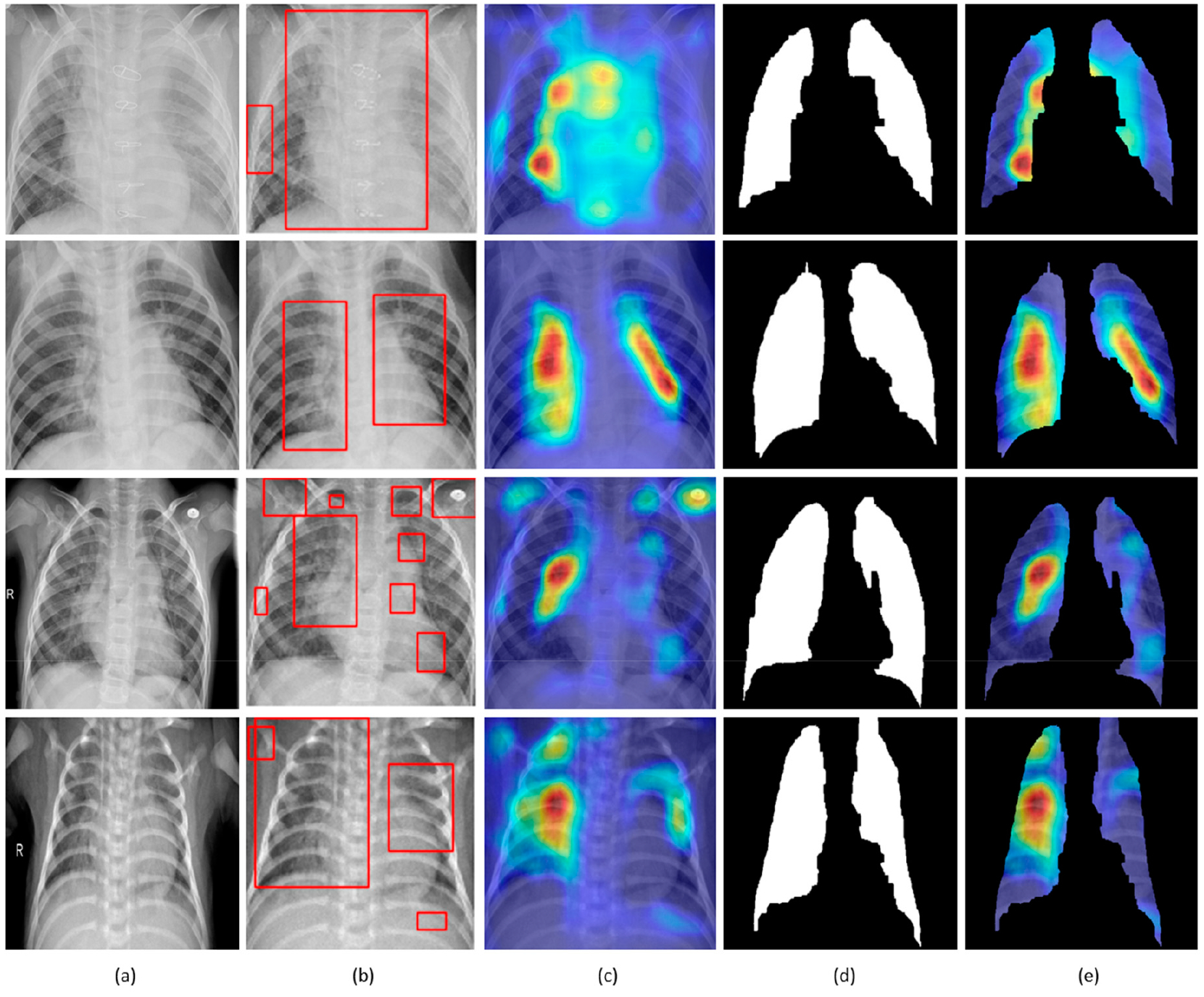
Visual explanations through gradient-based localization using grad-CAM: (**a**) Input CXRs; (**b**) Bounding boxes localizing regions of activations; (**c**) Grad-CAM showing heat maps superimposed on the original CXRs; (**d**) Automatically segmented lung masks; (**e**) Grad-CAM showing heat maps superimposed on the cropped lungs.

The customized VGG16 model has a CAM-compatible architecture owing to the presence of the GAP layer. This helps in visualizing the model predictions using both CAM and grad-CAM visualization tools. Figures 11 and 12 demonstrate the results of applying these visualizations to localize the discriminative ROI in pneumonia-infected CXRs.

CXRs are fed to the trained model and the predictions are decoded. The heat maps are generated as a two-dimensional score grid, computed for each input pixel location. Pixels carrying high importance with respect to the expected class appeared bright red with distinct color transitions for varying ranges. The generated heat maps are superimposed on the original input to localize image-specific ROI. The lung masks that are generated with the boundary detection algorithm are applied to extract the localized ROI relevant to the lung regions. We observed that CAM and grad-CAM visualizations generated heat maps for the pneumonia class to highlight the visual differences in the “pneumonia-like” regions of the image.

**Figure 13.**
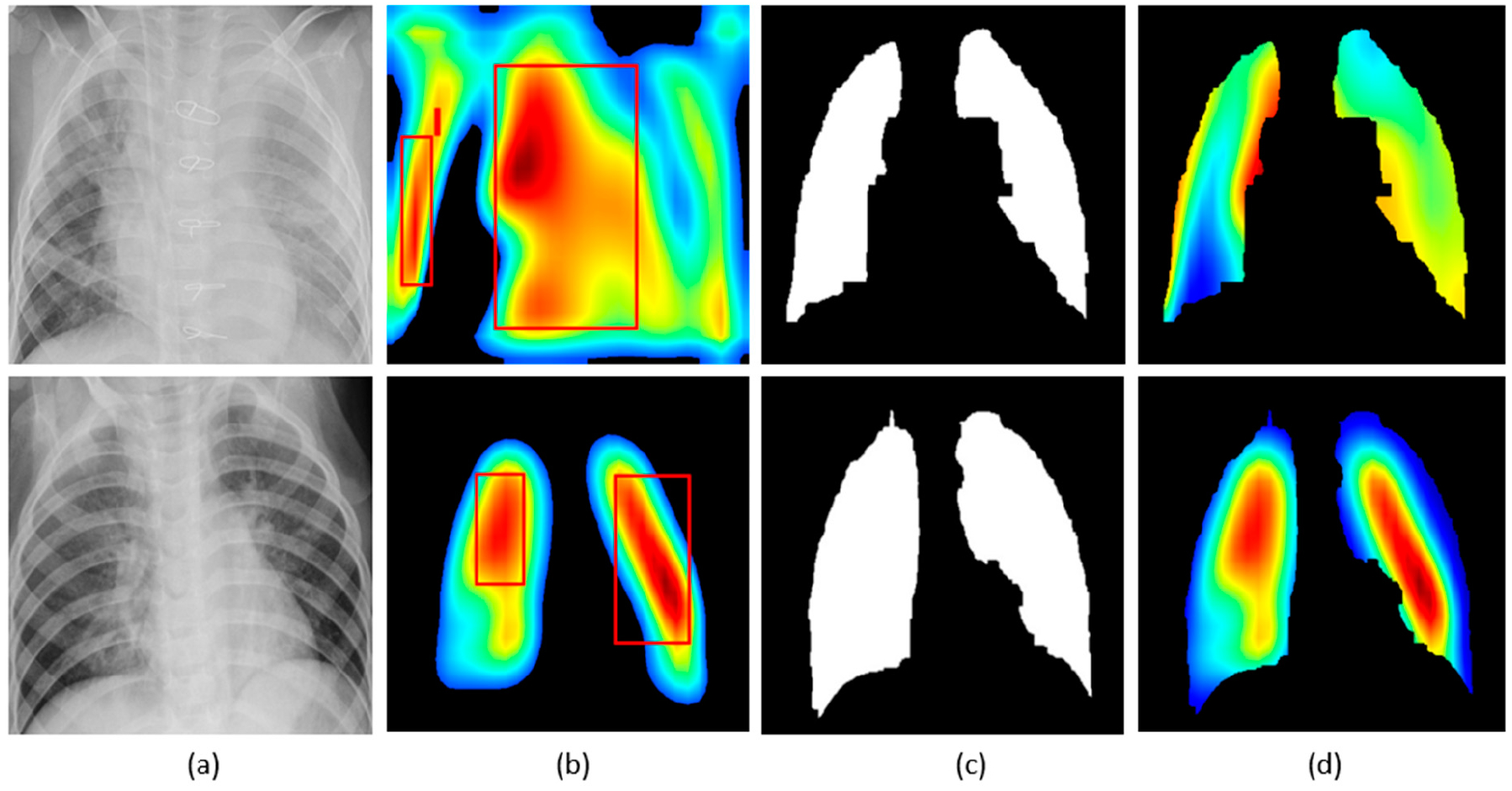
Visual explanations through average-CAM: (**a**) Bacterial and viral CXR (top and bottom); (**b**) Average-CAM localizing class-specific ROI with bounding boxes highlighting the regions of maximum activation; (**c**) Automatically segmented lung masks; (**d**) Average-CAM localizing class-specific ROI with the extracted lung regions.

**Figure 14.**
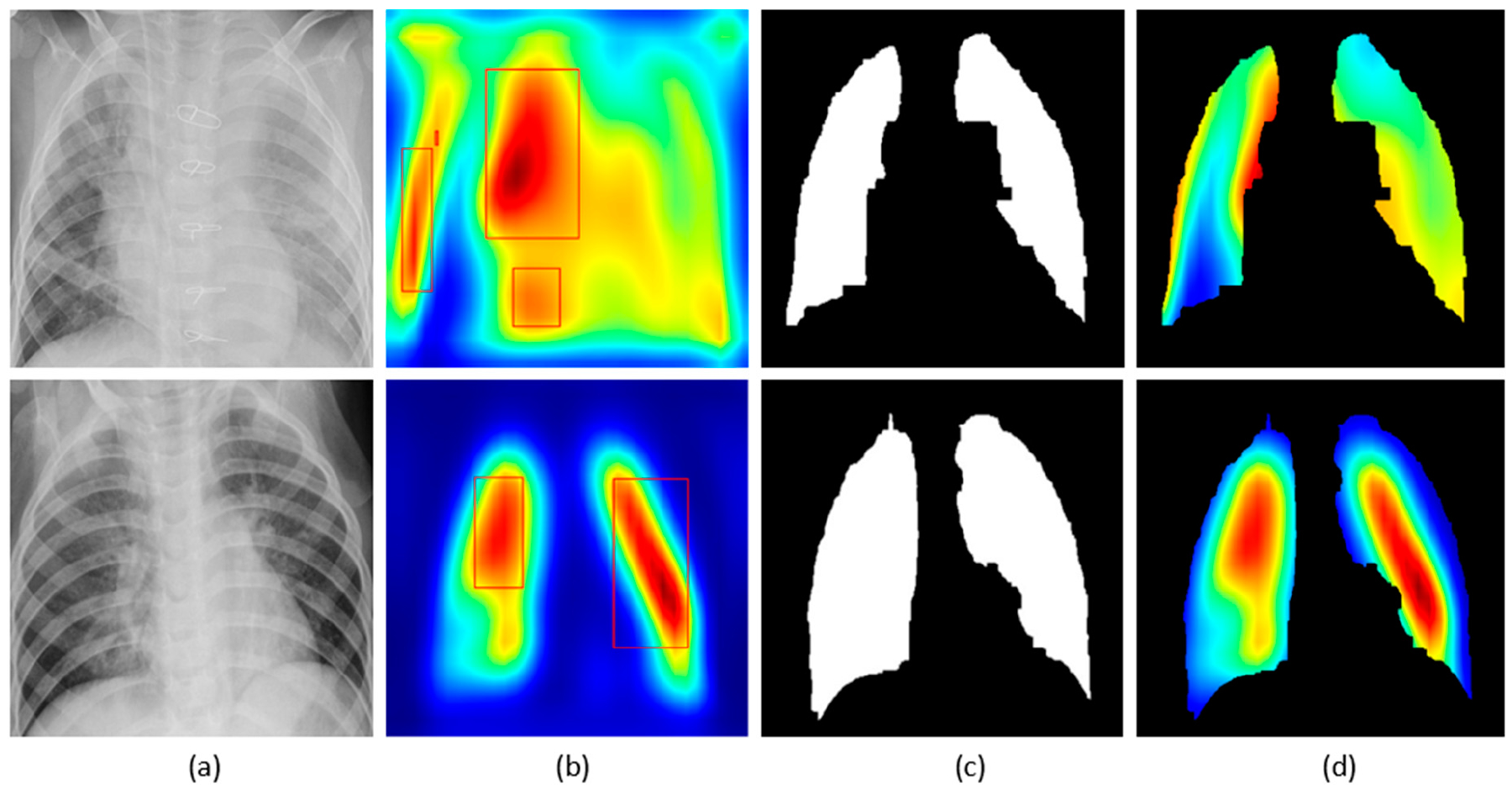
Visual explanations through average-grad-CAM: (**a**) Bacterial and viral CXR (top and bottom); (**b**) Average-grad-CAM localizing class-specific ROI with bounding boxes highlighting the regions of maximum activation; (**c**) Automatically segmented lung masks; (**d**) Average-grad-CAM localizing class-specific ROI with the extracted lung regions.

We applied our novel method of average-CAM and average-grad-CAM to visualize the class-specific ROI, as shown in Figures 13 and 14. Lung masks are applied to the generated heat maps to localize only the ROI specific to the lung regions. We observed that the class-specific ROI localized by the average-CAM and average-grad-CAM for the viral pneumonia class follows a diffuse pattern. This is obvious for the reason that viral pneumonia manifests with diffuse interstitial patterns in both lungs [30]. For the bacterial pneumonia class, we observed that the model layers are activated on both sides of the lungs, predominantly on the upper and middle right lung lobes. This is for the reason that bacterial pneumonia manifests as lobar considerations [30]. The pneumonia dataset under study has more pediatric patients with right lobar consolidations.

### 4.3. Visual Explanations with LIME

**Figure 15.**
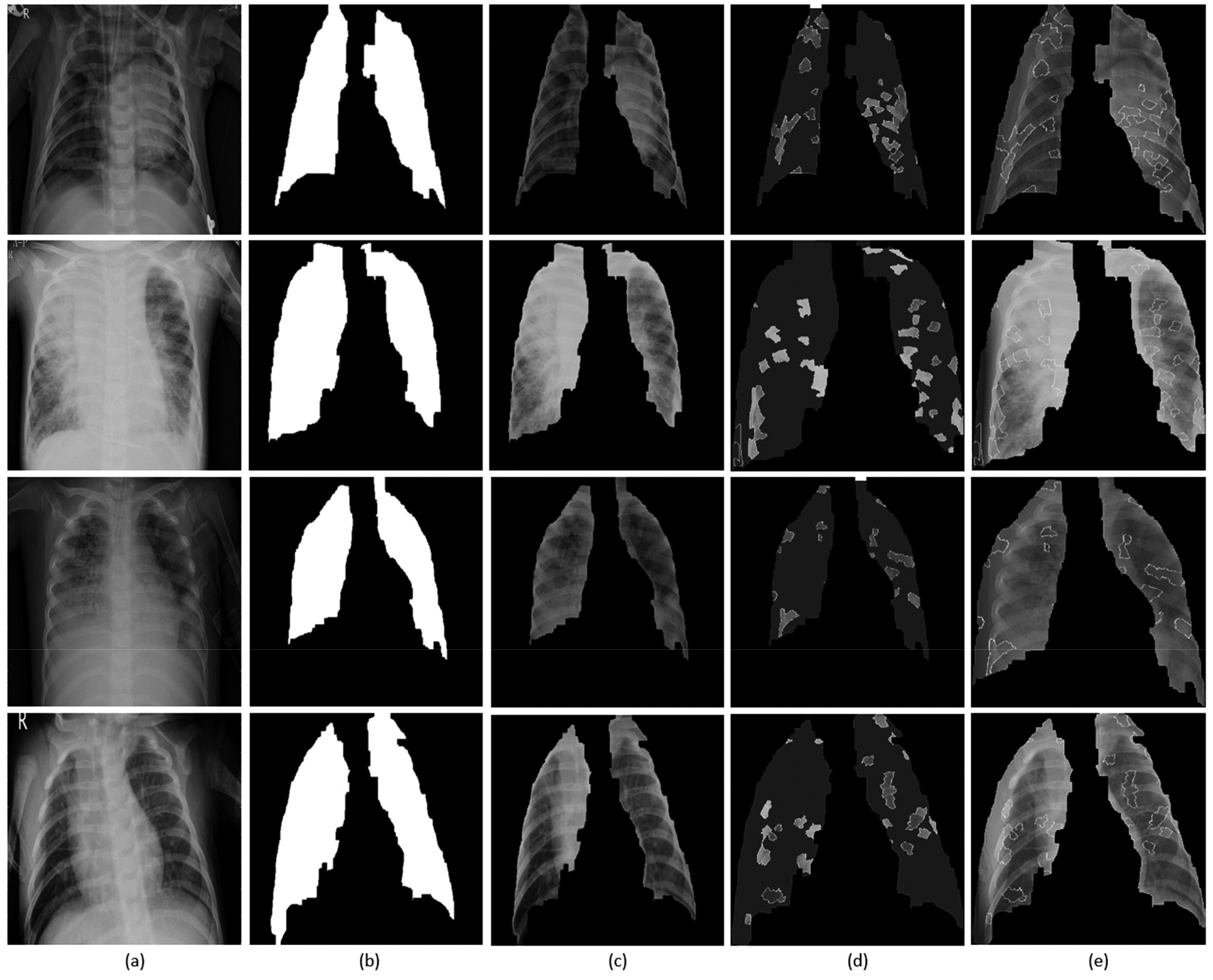
Visual explanations through LIME: (**a**) Input CXRs; (**b**) Automatically segmented lung masks; (**c**) Copped lung regions; (**d**) Superpixels with the highest positive weights with the others greyed out; (**e**) Superpixels with the highest positive weights are superimposed on the cropped lungs.

Figure 15 shows the explanations generated with LIME for sample instances of pediatric chest radiographs. Lung masks are applied to the explanations to localize only the ROI specific to the lung regions. The explanations are shown as follows:(i) Superpixels with the highest positive weights and the rest are greyed out; and, (ii) superpixels superimposed on the extracted lung regions. We observed that the explainer focused on the regions with high opacity. The model differentiates bacterial and viral pneumonia by (i) showing superpixels with the highest positive activations in the regions of lobar consolidations for bacterial pneumonia; and, (ii) diffuse interstitial patterns across the lungs for viral pneumonia. We also observed that a number of false positive superpixels are reported. The reason is that the current LIME implementation uses a sparse linear model to approximate the model behavior in the neighborhood of the explained predictions. However, these explanations result from a random sampling process and are not faithful if the underlying model is highly non-linear in the locality of predictions.

## 5. Conclusions

We proposed a CNN-based decision support system to detect pneumonia in pediatric CXRs to expedite accurate diagnosis of the pathology. We applied novel and state-of-the-art visualization strategies to explain model predictions that is considered highly significant to clinical decision-making. The study presents a universal approach to apply to an extensive range of visual recognition tasks. Classifying pneumonia in chest radiographs is a demanding task due to the presence of a high degree of variability in the input data. The promising performance of the customized VGG16 model trained on the current tasks suggest that it effectively learns from a sparse collection of complex data with reduced bias and improved generalization. We hope that our results are useful for developing clinically useful solutions to detect and distinguish pneumonia types in chest radiographs.

## Figures and Tables

**Table 1.** Dataset and its characteristics.

Category	Training Samples	Test Samples	File Type
Normal	1349	234	JPG
Bacterial	2538	242	JPG
Viral	1345	148	JPG

**Table 2.** Optimal values for the hyperparameters of the customized residual and inception CNNs obtained through a randomized grid search.

Model	Learning Rate	Momentum	L2 Regularization
Residual CNN	1 × 10^−3^	0.9	1 × 10^−6^
Inception CNN	1 × 10^−2^	0.95	1 × 10^−4^
Customized VGG16	1 × 10^−4^	0.99	1 × 10^−6^

**Table 3.** Performance of customized CNNs with baseline and cropped ROI data.

Task	Data	Models	Accuracy	AUC	Precision	Recall	Specificity	F-Score	MCC
Normal vs. Pneumonia	Baseline	Customized VGG16	**0.957**	**0.990**	**0.951**	**0.983**	**0.915**	**0.967**	**0.908**
		Sequential	0.943	0.983	0.920	0.980	0.855	0.957	0.878
		Residual	0.910	0.967	0.908	0.954	0.838	0.931	0.806
		Inception	0.886	0.922	0.887	0.939	0.800	0.913	0.755
	Cropped ROI	Customized VGG16	**0.962**	**0.993**	**0.977**	0.962	**0.962**	**0.970**	**0.918**
		Sequential	0.941	0.984	0.930	**0.995**	0.877	0.955	0.873
		Residual	0.917	0.971	0.913	0.959	0.847	0.936	0.820
		Inception	0.897	0.932	0.896	0.947	0.817	0.921	0.778
Bacterial vs. Viral Pneumonia	Baseline	Customized VGG16	**0.936**	**0.962**	**0.920**	**0.984**	**0.860**	**0.951**	**0.862**
		Sequential	0.928	0.954	0.909	**0.984**	0.838	0.946	0.848
		Residual	0.897	0.921	0.880	0.967	0.784	0.922	0.780
		Inception	0.854	0.901	0.841	0.934	0.714	0.886	0.675
	Cropped ROI	Customized VGG16	**0.936**	**0.962**	**0.920**	**0.984**	**0.860**	**0.951**	**0.862**
		Sequential	0.928	0.956	0.909	**0.984**	0.838	0.946	0.848
		Residual	0.908	0.933	0.888	0.976	0.798	0.930	0.802
		Inception	0.872	0.919	0.853	0.959	0.730	0.903	0.725
Normal vs. Bacterial vs. Viral Pneumonia	Baseline	Customized VGG16	**0.917**	**0.938**	**0.917**	**0.905**	**0.958**	**0.911**	**0.873**
		Sequential	0.896	0.922	0.888	0.885	0.948	0.887	0.841
		Residual	0.861	0.887	0.868	0.882	0.933	0.875	0.809
		Inception	0.809	0.846	0.753	0.848	0.861	0.798	0.688
	Cropped ROI	Customized VGG16	**0.918**	**0.939**	**0.920**	**0.900**	**0.960**	**0.910**	**0.876**
		Sequential	0.897	0.923	0.898	0.898	0.949	0.898	0.844
		Residual	0.879	0.909	0.883	0.890	0.941	0.887	0.825
		Inception	0.821	0.865	0.778	0.855	0.878	0.815	0.714

* Bold numbers indicate superior performance.

**Table 4.** Comparing the performance of the customized VGG16 model with the state-of-the-art.

Task	Model	Accuracy	AUC	Precision	Recall	Specificity	F-Score	MCC
Normal v. Pneumonia	Customized VGG16	**0.962**	**0.993**	**0.977**	**0.962**	**0.962**	**0.970**	**0.918**
	Kermany et al.	0.928	0.968	-	0.932	0.901	-	-
Bacterial v. Viral Pneumonia	Customized VGG16	**0.936**	**0.962**	**0.920**	**0.984**	**0.860**	**0.951**	**0.862**
	Kermany et al.	0.907	0.940	-	0.886	0.909	-	-
Normal v. Bacterial v. Viral Pneumonia	Customized VGG16	**0.918**	**0.939**	**0.920**	**0.900**	**0.960**	**0.910**	**0.876**
	Kermany et al.	-	-	-	-	-	-	-

* Bold numbers indicate superior performance.
